# Stereoselective Voltammetric Biosensor for Myo-Inositol and D-Chiro-Inositol Recognition

**DOI:** 10.3390/s23229211

**Published:** 2023-11-16

**Authors:** Cristina Tortolini, Valeria Gigli, Flavio Rizzo, Andrea Lenzi, Mariano Bizzarri, Antonio Angeloni, Riccarda Antiochia

**Affiliations:** 1 Department of Experimental Medicine, Sapienza University of Rome, Viale Regina Elena 324, 00161 Rome, Italy; cristina.tortolini@uniroma1.it (C.T.); valeria.gigli@uniroma1.it (V.G.); flavio.rizzo@uniroma1.it (F.R.); andrea.lenzi@uniroma1.it (A.L.); mariano.bizzarri@uniroma1.it (M.B.); antonio.angeloni@uniroma1.it (A.A.); 2 Department of Chemistry and Drug Technologies, Sapienza University of Rome, Piazzale Aldo Moro 5, 00185 Rome, Italy

**Keywords:** myo-inositol, D-chiro-inositol, biosensor, stereoselective recognition, voltammetric detection, MWCNTs

## Abstract

This paper describes the development of a simple voltammetric biosensor for the stereoselective discrimination of myo-inositol (myo-Ins) and D-chiro-inositol (D-chiro-Ins) by means of bovine serum albumin (BSA) adsorption onto a multi-walled carbon nanotube (MWCNT) graphite screen-printed electrode (MWCNT-GSPE), previously functionalized by the electropolymerization of methylene blue (MB). After a morphological characterization, the enantioselective biosensor platform was electrochemically characterized after each modification step by differential pulse voltammetry (DPV) and electrochemical impedance spectroscopy (EIS). The results show that the binding affinity between myo-Ins and BSA was higher than that between D-chiro-Ins and BSA, confirming the different interactions exhibited by the novel BSA/MB/MWCNT/GSPE platform towards the two diastereoisomers. The biosensor showed a linear response towards both stereoisomers in the range of 2–100 μM, with LODs of 0.5 and 1 μM for myo-Ins and D-chiro-Ins, respectively. Moreover, a stereoselectivity coefficient α of 1.6 was found, with association constants of 0.90 and 0.79, for the two stereoisomers, respectively. Lastly, the proposed biosensor allowed for the determination of the stereoisomeric composition of myo-/D-chiro-Ins mixtures in commercial pharmaceutical preparations, and thus, it is expected to be successfully applied in the chiral analysis of pharmaceuticals and illicit drugs of forensic interest.

## 1. Introduction

Myo-inositol (myo-Ins) and D-chiro-inositol (D-chiro-Ins) are C_6_ sugar alcohols belonging to the large inositol family [1]. Among the nine stereoisomers of the inositol family, myo-Ins is the predominant physiological form. There is a proper myo-Ins/D-chiro-Ins ratio content for each body organ and tissue, which is necessary to maintain healthy conditions. For instance, the myo-/D-chiro-Ins plasma ratio in healthy women is 40:1, whereas in ovarian follicular fluid, it is close to 100:1 [2]. Both isomers are involved in the glycemic regulation exerting insulin-mimetic activity and being effective against insulin resistance [3,4,5,6,7,8,9,10]. Moreover, myo-Ins exhibits an important role in the phosphatidyl-inositol cycle, an important intracellular signaling pathway [11], and it has been observed that myo-Ins depletion may expose patients to several neuropsychiatric conditions, such as Alzheimer’s and Parkinson’s diseases, depression, and amyotrophic lateral sclerosis, thus assuming a possible protective role of myo-Ins in several neurodegenerative and neurological disorders [12].

The endogenous production of myo- and D-chiro-Ins stereoisomers strongly varies depending on tissue needs. The myo-Ins concentration in adults is generally between 13 and 43 μM [1], while that of D-Chiro-Ins is between 0.15 and 0.90 μM [13] and 0.33 and 9.8 μM in men and women [14], respectively.

In addition to glycemic control, myo- and D-chiro-inositol play an essential role in female and male reproduction physiology [14]. As reported above, the ovaries of healthy women are characterized by a myo-/D-chiro-Ins ratio of about 100:1, which corresponds to optimum physiological conditions. However, the ovaries of polycystic ovary syndrome (PCOS) patients show an opposite ratio, with an increase in D-chiro-Ins and a depletion of myo-Ins content [15,16,17,18,19,20,21,22,23,24,25].

Therefore, altered inositol levels or abnormalities in their metabolism may have direct implications for several neurological, hormonal, and reproductive disorders. To this aim, inositol therapy can should be utilized to restore the inositol physiological ratio or to alter this ratio in order to achieve specific effects. It has been found that the combined therapy of myo-/D-chiro-Ins reproducing the plasma physiological ratio (40:1) allows for the achievement of better clinical results, in particular in improving the endocrine and metabolic parameters in young overweight women affected by PCOS.

Thus, a sensitive and accurate method for selective discrimination between myo-Ins and D-chiro-Ins stereoisomers could be of great interest in the field of pharmaceutical science and biochemistry [26]. Generally, the stereoselective determinations are based on chromatographic [27,28,29] and electromigration methods [30,31], which use chiral stationary phases and chiral selectors, respectively. Similar chiral selectors can be utilized in electrochemical sensors and biosensors for the enantio/stereoselective determination of a particular isomer [32,33,34,35], showing the merits of simple, rapid, low-cost, disposable, and sensitive detection. Only a few chiral sensors have been reported in the literature based on the use of different nanomaterials for the enantioselective discrimination of enantiomers, such as propranolol [36], tryptophan [37,38], vasopressin [39], mandelic acid [40,41], penicillamine [42], and naproxen [43]. At present, human serum albumin (HSA) and bovine serum albumin (BSA) show the highest potential stereoselectivity among all plasma proteins [44]. They are known to possess a unique capability to bind a great variety of endogenous and exogenous compounds, essentially through hydrophobic, steric, and electrostatic interactions [45,46], which are the basis of the affinity biosensor mechanism [47,48,49,50]. The enantioselectivity is generally demonstrated by cyclic voltammetry (CV), differential pulse voltammetry (DPV), and electrochemical impedance spectroscopy (EIS) using a redox probe of [Fe(CN)]_6_^4−^/^3−^ in the detecting solution [38].

The aim of this work is to realize a reagentless voltammetric biosensor by immobilizing bovine serum albumin (BSA) onto a methylene/multi-walled nanotube (MB/MWCNT) modified graphite screen printed electrode (GSPE) for the stereoselective discrimination of myo-Ins and D-chiro-Ins. To the best of our knowledge, this is the first time that a chiral sensor has been developed for the discrimination of these two stereoisomers. The stereoselectivity of the BSA/MB/MWCNT/GSPE platform was studied by varying BSA concentrations, binding times, and myo-Ins and D-chiro-Ins concentrations. Discrimination was due to the different irreversible binding of the two stereoisomers to BSA molecules, investigated also by molecular docking studies. The proposed biosensor was utilized for the determination of the percentage ratio of myo-Ins in a stereoisomeric mixture, with important potential applications in chiral drug analysis.

## 2. Materials and Methods

### 2.1. Reagents

Myo-inositol (myo-Ins), D-chiro-inositol (D-chiro-Ins), methylene blue (MB), bovine serum albumin (BSA), human serum albumin (HSA), multiwall carbon nanotubes (MWCNTs, d = 110–170 nm, length = 5–9 micron, 90+%), ethanol, potassium ferricyanide (III) (K_3_[Fe(CN)_6_]), potassium ferrocyanide (II) (K_4_[Fe(CN)_6_]), sodium phosphate monobasic (NaH_2_PO_4_), sodium phosphate dibasic (Na_2_HPO_4_), and potassium chloride (KCl) were obtained from Sigma Aldrich (Bucks, Germany). All solutions were prepared in phosphate buffer 50 mM, KCl 0.1 M, pH 7.0 (PBS). High-purity deionized water (resistance: 18.2 MΩ cm at 25 °C; TOC < 10 µg L^−1^) obtained from Millipore (Molsheim, France) was used throughout all of the experiments.

### 2.2. Apparatus

Scanning electron microscopy (SEM) and energy-dispersive X-ray spectroscopy analysis (EDX) experiments were carried out with high-resolution field emission scanning electron microscopy (HR FESEM, Zeiss Auriga Microscopy, Jena, Germany) for the characterization of surface-modified electrodes. Cyclic voltammetry (CV), differential pulse voltammetry (DPV), and electrochemical impedance spectroscopy (EIS) were performed with an Autolab PGSTAT204 potentiostat, operated by Nova v 2.1 (Metrohm, Herisau, Switzerland) using a three-electrode system consisting of a graphite screen-printed electrode (DRP-110, d = 4 mm Metrohm Italiana, Oreggio, Italy, GSPE) as a bare or modified working electrode, a carbon rod as a counter-electrode, and silver/silver chloride (Ag/AgCl, 1 M KCl) as a reference electrode. 

Raman measurements were recorded using the iRaman plus (B&WTek) model BWS465-785S with a wavelength of 785 nm, with a laser power at the probe of 340 mW and using a High Quantum Efficiency CCD Array detector (Metrohm Italiana S.r.l., Origgio (VA), Italy).

### 2.3. Nanobiosensor Fabrication Steps

**Scheme 1:**
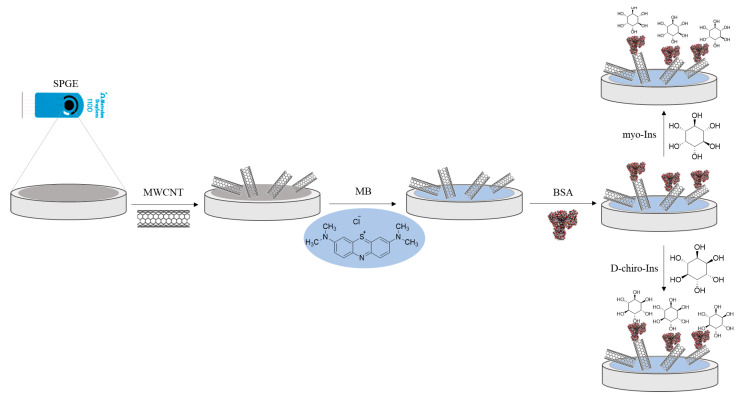
Schematic representation of myo-Ins/D-chiro-Ins enantioselective biosensor fabrication steps and sensing mechanism.

The BSA/MB/MWCNT/GSPE and HSA/MB/MWCNT/GSPE nanobiosensors were prepared according to the following steps: (i) 10 μL of the MWCNT solution (10 mg mL^−1^ in a solution of water/ethanol 70:30) was drop-cast onto GSPE and left to dry at room temperature for 30 min; (ii) MWCNT/GSPE was modified by electropolymerization of MB 0.1 mM in PBS 50 mM, pH 7.0, KCl 0.1 M by cyclic voltammetry from −1.0 V to +1.0 V, at ν of 100 mV s^−1^, for 20 scan; (iii) 10 μL of BSA or HSA solution (0.1 mg mL^−1^ in PBS 50 mM, pH 7.0, KCl 0.1 M) was drop-cast onto GSPE previously modified and left to dry at room temperature for 30 min. Scheme 1 shows the modified electrode fabrication steps and the sensing mechanism towards myo-Ins and D-chiro-Ins.

### 2.4. Electrochemical Measurements

Electrochemical characterization of bare and modified GSPE was performed with the CV, DPV, and EIS techniques in 0.1 M KCl solution containing 5 mM [Fe(CN)_6_]^3−/4−^ as an electrochemical redox probe (Zobell’s solution). In particular, for the CV measurements, a potential range from −0.2 to 0.7 V (vs. Ag/AgCl, reference electrode) and a scan rate of 25 mV s^−1^ were used. EIS data were run in the frequency range of 0.01–10^3^ Hz, using an AC signal of 0.01 V amplitude and under open-circuit potential (OCP) conditions.

The sensing capabilities of MB/MWCNT/GSPE by using BSA towards myo-Ins and D-chiro-Ins and HSA towards myo-Ins were evaluated using the DPV technique. The measurements were carried out using 50 μM myo-Ins in 50 mM PBS, pH 7.4, in a potential range from −0.4 to 0.6 V (vs. Ag/AgCl reference electrode) under the following conditions: 20 mV pulse amplitude and 4 mV E step. The optimization studies for the final sensor and the calibration plots were carried out under the same DPV conditions. All measurements were performed in triplicate.

### 2.5. Molecular Docking

The structure of the BSA (PDB id: 4F5S) was obtained by X-ray diffraction and used as a template [51]. The structures of myo-Ins, D-chiro-Ins, and folic acid were obtained by ChemSpider (Royal Society of Chemistry). Images of the structures were generated using Pymol (DeLano Scientific, Palo Alto, CA, USA). The docking runs were performed on the HDock docking server [52]. The whole protein, BSA, was selected as a potential binding site of the three compounds. Among the several models, the best of each one based on the docking score was selected for further calculations. The Prodigy web server [53] was utilized to calculate the binding affinity of predicted protein–ligand complexes.

### 2.6. Analysis of Pharmaceutical Preparations

Four commercial pharmaceutical preparations were obtained from a local pharmacy and dissolved into 100 mL of PBS 50 mM, pH 7.0, under continuous stirring for 5 min.

The solutions were filtered with filter paper and successively diluted to 1000-fold with PBS 50 mM, pH 7.0, before the electrochemical experiments.

## 3. Results and Discussion

### 3.1. Characterization of the Different Electrode Surfaces

#### 3.1.1. SEM and EDX

**Figure 1:**
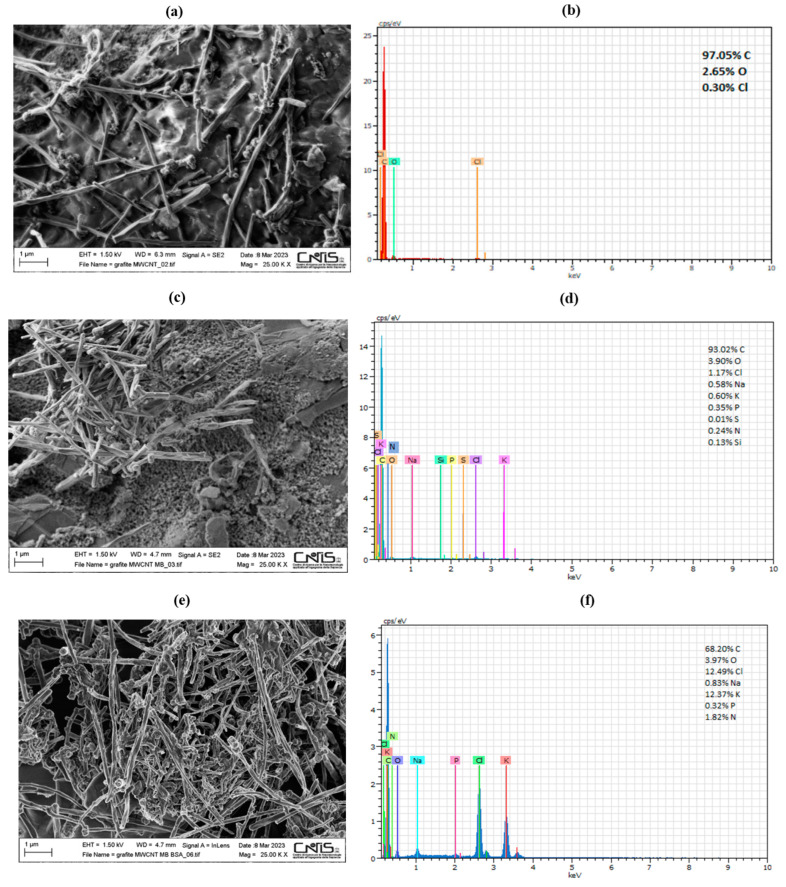
SEM images and EDX spectra for: MWCNT/GSPE (**a**,**b**); MB/MWCNT/GSPE (**c**,**d**) and BSA/MB/MWCNT/GSPE (**e**,**f**). Experimental conditions: magnification: 25 K X; voltage: 1.50 kV.

The surface morphology of the modified SPEs was initially analyzed by SEM and EDX analysis. The SEM images of MWCNT/GSPE, MB/MWCNT/GSPE, and BSA/MB/MWCNT/GSPE are shown in Figure 1, while the bare GSPE is reported in Figure S1 (Supplementary Materials), for comparison. The MWCNT film appears as a layer of uniform tubes with a diameter of a few nanometers (Figure 1a), and the EDX spectra (Figure 1b) confirm atomic percentages of carbon (97%) and oxygen (~3%). The low amount of Cl element (<1%) can be ascribed to the presence of possible impurities in the commercial GSPE.

After MB electropolymerization, a fairly uniform layer of polymer was visible on the MB/MWCNT/GSPE electrode surface (Figure 1c). The related EDX spectra (Figure 1d) confirm the presence of N and S atoms due to the occurrence of polymerization of MB on the biosensor surface. Lastly, the SEM image of the BSA/MB/MWCNT/GSPE clearly shows the presence of a tick fibrous macro-structure, which indicates the immobilization of the BSA biomolecule, which properly covers the electrode surface, thanks to its high abundance of charged amino acid, which provides it with great adsorptive power (Figure 1e). The corresponding EDX mapping reveals an increased amount of N and several heteroatom (K and Na) peaks, indicating the effective binding of the protein that occurred on the nanomodified surface (Figure 1f). The chemical composition of the platform is consistent with sodium and potassium chloride (NaCl, KCl), which was used for the commercial lyophilized BSA powder preparation.

#### 3.1.2. Raman Spectroscopy

The Raman spectra of bare graphite and MWCNT/GSPE are reported in Figure S2 (Supplementary Materials). Both spectra show two unambiguous peaks at around 1650 and 1360 cm^−1^, corresponding to the typical G and D peaks of carbon materials, due to the bond stretching of all pairs of sp^2^ atoms in both rings and chains, and to the breathing modes of sp^2^ atoms in the rings, respectively.

### 3.2. Electrochemical Characterization of the MWCNT Electrochemical Platform

**Figure 2:**
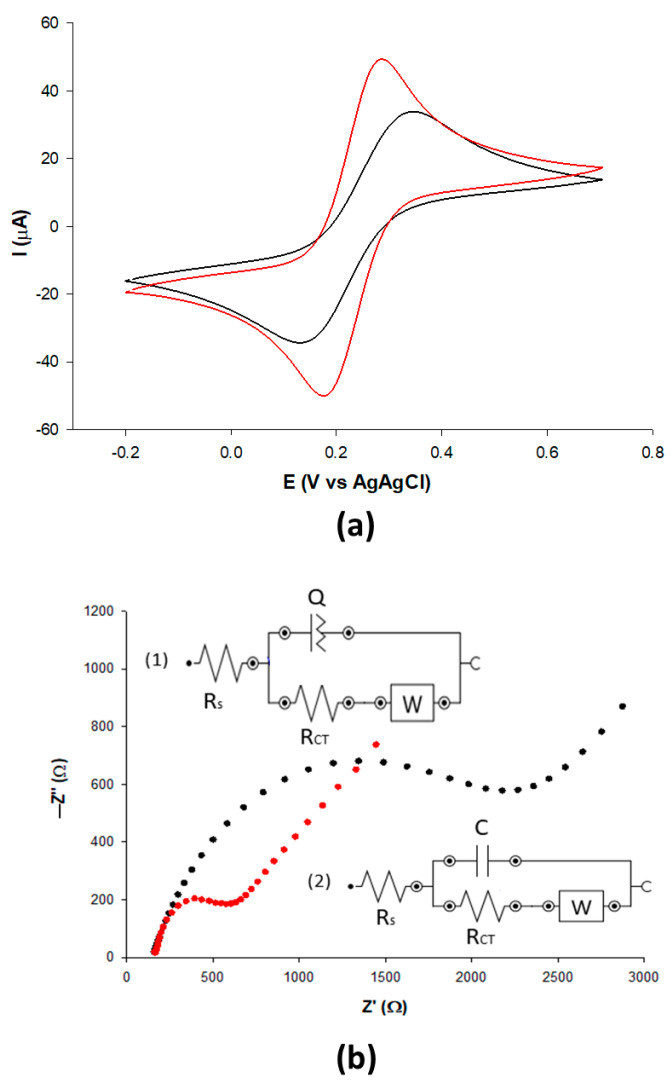
CVs (**a**) and EIS spectra (**b**) of bare/SPGE (black line) and MWCNT/GSPE (red line). Redox probe: 5 mM [Fe(CN)_6_]^3−/4−^ containing 0.1 M KCl solution. Scan rate: 25 mV s^−1^. Inset: (1) [R(Q[RW])] and (2) [R(C[RW])] circuits used for fitting the experimental data.

The electrochemical behavior of the GSPE before and after functionalization with MWCNTs was investigated by CV and EIS experiments. Figure 2a shows the CVs obtained before (black curve) and after the modification with MWCNTs (red curve), performed in Zobell’s solution. Compared to bare electrodes, which showed an irreversible behavior (black curve), the CV obtained with MWCNT/GSPE (red curve) showed a couple of quasi-reversible redox peaks, with a ΔE_p_ value of 105 mV, very close to the value of a perfect two-electron reversible electron process (Table 1). The peak current showed an opposite trend, with the lower peak current value obtained with the bare electrode (black curve), and the higher value with the MWCNT modified electrode (red curve), thanks to the larger surface area and the excellent conductivity of the MWCNTs. Table 1 shows the electroactive area (A_e_), the roughness factor (*ρ*), and the heterogeneous electron transfer rate constants (k^0^, cm s^−1^) of the two electrodes. The A_e_ was determined by calculating the slope of the I_p_ vs. *v*^1/2^ plot and successively inserting this value into the following Randles–Sevcik equation [54]:

I_p_ = 2.686 × 10^5^ n^3/2^ A_e_ D_0_^1/2^ C_0_ *v*^1/2^ (1)

where I_p_ = voltammetric peak current (A), n = number of electrons, A_e_ = electroactive area (cm^2^), D_0_ = diffusion coefficient (7.6 × 10^−6^ cm^2^ s^−1^ for ferricyanide), C_0_ = concentration (mol cm^−3^), *v* = scan rate (V s^−1^), and the roughness factor (*ρ*) as the ratio of the electroactive/geometric area. The cyclic voltammograms relative to the electrode platforms recorded at different scan rates are shown in Figure S3 (Supplementary Materials). The heterogeneous electron transfer rate constants (k^0^, cm s^−1^) for the GSPE before and after the modification with MWCNTs were calculated with the method of Lavagnini et al., which merges an irreversible (Klingler–Kochi) and a reversible system method (Nicholson and Shain) [55]. The highest k^0^ value (k^0^ = 1.66 ± 0.14 cm s^−1^) obtained with the MWCNT/GSPE electrode confirms the faster electron transfer realized by the nanostructured MWCNTs.

EIS was utilized to study the changes in the interfacial features during the different modification steps of the proposed sensor. Figure 2b shows the Nyquist plot for the bare GSPE (black curve) and the MWCNT modified GSPE (red curve), using [Fe(CN)_6_]^3−/4−^ as a redox probe. The high-frequency region spectra, which represent the charge transfer (R_CT_) and charge separation (R_S_) resistance, were analyzed in more detail (Table 2). The R_CT_ values of the modified electrode decreased significantly from about 2000 to ∼500 Ω, compared to the bare electrode. This result highlights the importance of the employment of the nanomaterial for a clear improvement in sensor conductivity after the modification with MWCNTs and confirms the results obtained by the CV technique. The impedance spectra were fitted using two equivalent circuits, a simple Randles circuit [R(Q[RW])] for the bare electrode (inset in Figure 2, circuit 1), and a Randles equivalent circuit, [R(C[RW])], for the MWCNTs and successively modified electrodes (inset in Figure 2, circuit 2).

### 3.3. Electrochemical Characterization of the Stereoselective Biosensor Platform

**Figure 3:**
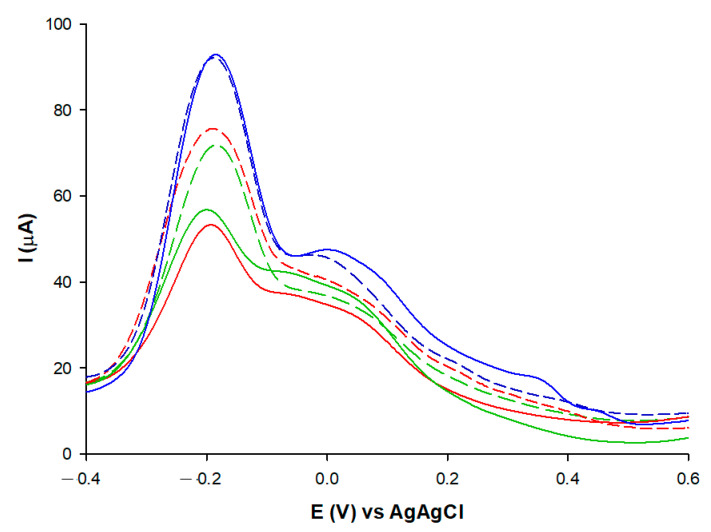
DPV in PBS 50 mM, pH 7.0 of MB/MWCNT/GSPE (blue line), BSA/MB/MWCNT/GSPE (red line), HSA/MB/MWCNT/GSPE (green line) in absence (dotted line) and in presence (continuous line) of 50 μM myo-Ins. Incubation time: 15 min.

The electrochemical MWCNT/GSPE behavior after each surface modification step for the construction of the myo-/D-chiro-Ins stereoselective biosensor was investigated using DPV experiments (Figure 3).

In order to develop a probe-less biosensor, the MWCNT/GSPE was initially modified by the electrodeposition of MB, as described in Section 2.3. Two redox peaks are clearly visible at −0.2 and 0.0 V, respectively, corresponding to the formation of the polymeric film [56] (Figure 3, dotted blue curve). In particular, the peak registered at −0.2 V shows a large peak current, due to the extraordinary electroactive properties of MB. Then, a peak current progressive decrease is observed in correspondence to the following electrode modification steps: (i) drop-casting of BSA (dotted red curve); (ii) drop-casting of HSA (dotted green curve); (iii) formation of myo-Ins/BSA complex (red curve); (iv) formation of myo-Ins/HSA complex (green curve). The results obtained clearly indicate that the myo-Ins was strongly adsorbed onto both the BSA and HSA molecules through non-covalent electrostatic interactions, and therefore, the electron transfer through the electrode surface was hindered by the insulating nature of myo-Ins. The lower current registered with the myo-Ins/BSA complex indicates a larger affinity of myo-Ins for BSA than HSA, and therefore, BSA was used for further experiments.

The reproducibility of the BSA/MB/MWCNT/GSPE platform was tested by repeatedly measuring the current in a PBS solution, pH=7.0, in the presence of a fixed myo-Ins amount, and the peak current remained at 97% of its initial value, confirming its excellent reproducibility (RSD = 3.0%, *n* = 10).

It must be observed that the DPV curve registered at the MB/MWCNT/GSPE without modification of the BSA or HSA showed no significative difference when the myo-Ins was added to the solution (Figure 3, blue curve), confirming that in the absence of both BSA and HSA molecules, myo-Ins was not adsorbed to the electrode surface.

**Figure 4:**
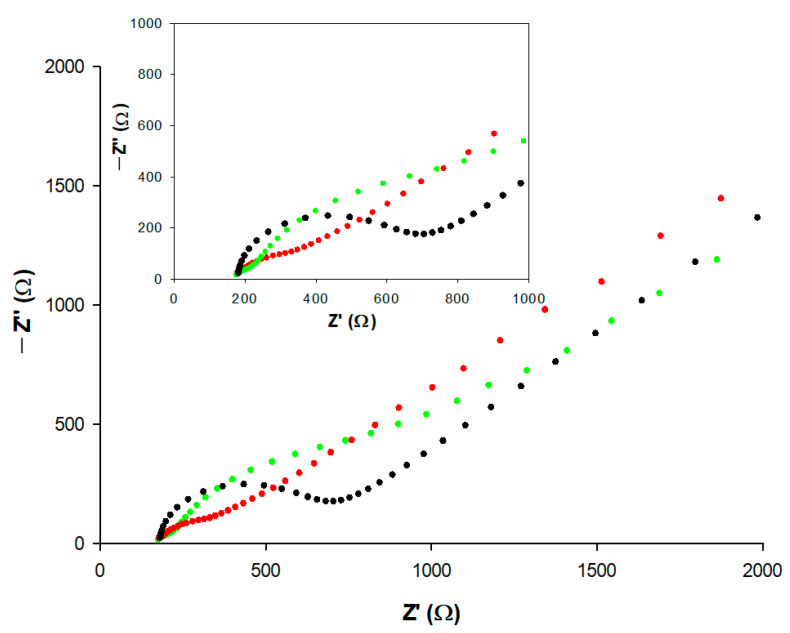
Nyquist’s plots of MWCNT/GSPE (black curve), MB/MWCNT/GSPE (red curve), and BSA/MB/MWCNT/GSPE (green curve). Redox probe: 5 mM [Fe(CN)_6_]^3−/4−^ containing a 0.1 M KCl solution.

An EIS study was also carried out to shed light on the different modification steps. Impedance spectra were recorded in Zobell’s solution at a fixed potential of 0.22 V vs. Ag/AgCl (Figure 4). The semicircle of the Nyquist plot initially decreased after modification with MB (red curve), then increased after the immobilization of the non-conductive BSA molecule (green curve) and the further addition of myo-Ins (blue curve). The fitting parameters obtained by the Randles equivalent circuit are reported in Table 2. It is interesting to note that the capacitance value (C) shows an opposite trend with an initial increase after the addition of MB polymer, due to an increase in the electroactive area of the electrode, showing a faster electron transfer in the presence of MWCNTs within the MB modified film and a successive decrease after BSA immobilization (Table 2).

### 3.4. Optimization of the BSA/MB/MWCNT/GSPE Platform

Preliminary experiments were dedicated to the optimization of the BSA concentration, which affected the electrochemical properties of the modified electrode, and therefore, the chiral recognition. The amount of BSA cast on the MB/MWCNT/GSPE was evaluated to be from 2 to 14 μL (Figure S4a, Supplementary Materials). The current peak values initially increased at increasing BSA concentrations and then reached a plateau for the affinity-based sensor prepared from 10 to 14 μL BSA, thus indicating the saturation of the electrode surface with BSA molecules. Thus, 10 μL was chosen as the optimum BSA amount for the fabrication of the proposed biosensor.

Successively, the effect of the BSA binding time in the range of 1-30 min was tested (Figure S4b, Supplementary Materials). The current signal increased from 1 to 15 min. A later enhancement in time resulted in no further current increases. Therefore, 15 min was adopted as the best incubation time for BSA immobilization for further studies.

### 3.5. Stereoselective Responses of Myo-Ins and D-Chiro-Ins on BSA/MB/MWCNT/GSPE Platform

DPV was used to study the enantioselective responses of myo-Ins and D-chiro-Ins on the BSA/MB/MWCNT/GSPE platform.

**Figure 5:**
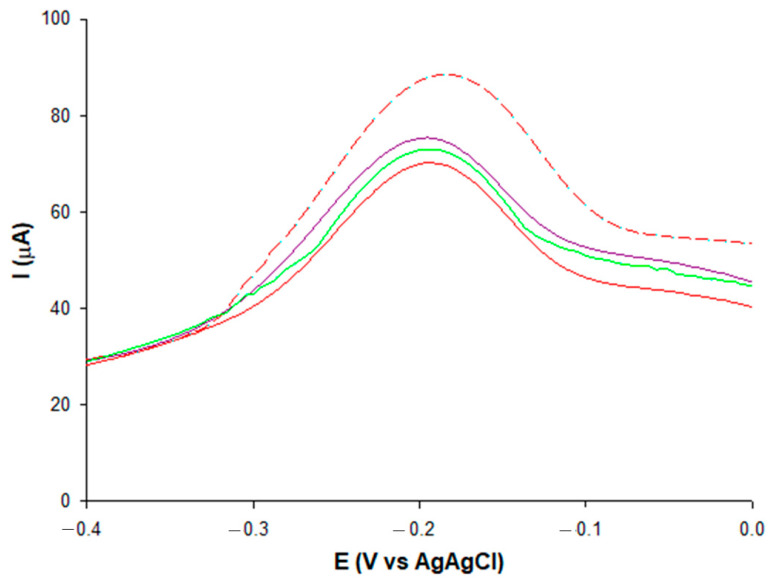
DPV of BSA/MB/MWCNT/GSPE in PBS 50 mM, pH 7.0, in absence (dotted red curve) and in presence (continuous line) of 50 μM myo-Ins (red curve), 50 μM D-chiro-Ins (violet curve), and a mixed solution of 25 mM myo-Ins and 25 mM D-chiro-Ins (green curve). Incubation time: 15 min.

Figure 5 shows the DPV peak current decrease after the incubation of the BSA/MB/MWCNT/GSPE in a 50 μM myo-Ins (continuous red curve) and D-chiro-Ins (continuous violet curve) solution for 15 min. A significative difference was observed, as the peak current decreased by ~15 μA after the interaction with myo-Ins, while a smaller decrease was registered in the presence of D-chiro-Ins (~10 μA). An intermediate decrease was registered with a mixed solution of equal concentrations of myo-Ins and D-chiro-Ins (continuous green curve). These results clearly indicate better adsorption of myo-Ins compared to D-chiro-Ins to BSA molecules, which causes a greater electron transfer hindrance of MB to the electrode surface. This fact may be ascribed to the lower hydrophobic nature of myo-Ins compared to the D-chiro-Ins molecule, which allows for stronger binding to the BSA hydrophilic cavities. The results suggest a higher binding affinity of myo-Ins to BSA, with consequent faster interaction kinetics, compared to D-chiro-Ins, due to the specific molecular configuration of myo-Ins, which better matches with BSA molecules through van der Waals interactions and H-bonding [46].

The stereoselectivity of the chiral sensor was determined quantitatively using the stereoselectivity coefficient α, defined as follows [38]:

α = ΔI_L_/ΔI_D_ (2)

where ΔI_L_ and ΔI_D_ are the differences in the peak current responses of the myo-Ins and D-chiro Ins solutions, respectively, and α represents a quantitative measure of the chiral sensor’s capacity to discriminate between stereoisomer molecules. The stereoselectivity of our sensor resulted in a value of 1.63.

### 3.6. Electrochemical Response at Varying Myo-Ins and D-Chiro-Ins Concentrations

**Figure 6:**
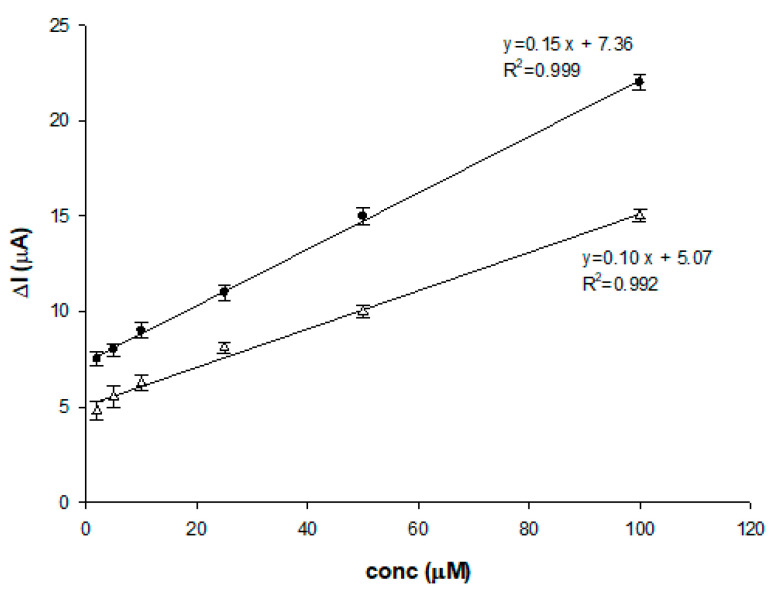
Calibration curves of BSA/MB/MWCNT/SPGE at different myo-inositol (●) and D-chiro-inositol (Δ) concentrations.

The sensing performance of the BSA/MB/MWCNT/GSPE for myo-Ins and D-chiro-Ins was investigated. Figure 6 shows the peak current responses to myo-Ins (ΔI_M_) and D-chiro-Ins (ΔI_D_) at various concentrations. It clearly appears that ΔI_M_ were always higher than ΔI_D_, with a gradual increase in the difference between ΔI_M_ and ΔI_D_ at increasing concentrations of both stereoisomers. The calibration plots for both isomers show a linear range between 2 and 100 μM, with a sensitivity of 0.15 μA μM^−1^ (R^2^ = 0.999) for myo-Ins, and a slightly lower sensitivity for D-chiro-Ins (0.10 μA μM^−1^, R^2^ = 0.992). The detection limits were found to be 0.5 and 1 μM for myo-Ins and D-chiro-Ins, respectively, calculated with the formula 3σ/S, where σ is the standard deviation of the intercept, and S is the slope of the calibration plot. These results clearly indicate that the BSA/MB/MWCNT/GSPE biosensor can be used for the chiral recognition of myo-/D-chiro-Ins with a better affinity for myo-Ins due to an easier adsorption of myo-Ins to the BSA-modified platform.

The association constant (*k*) and binding number (m) values between BSA and myo-Ins and D-chiro-Ins were obtained using the following equation [57]:

log(ΔI/ΔI_max_ − ΔI) = log*k* + m log[Ins] (3)

where ΔI is the peak current difference before and after the interaction of BSA/MB/MWCNT/GSPE with myo-Ins or D-chiro-Ins, ΔI_max_ is the maximum peak current variation, and [Ins] is the concentrations of the Ins stereoisomers.

**Figure 7:**
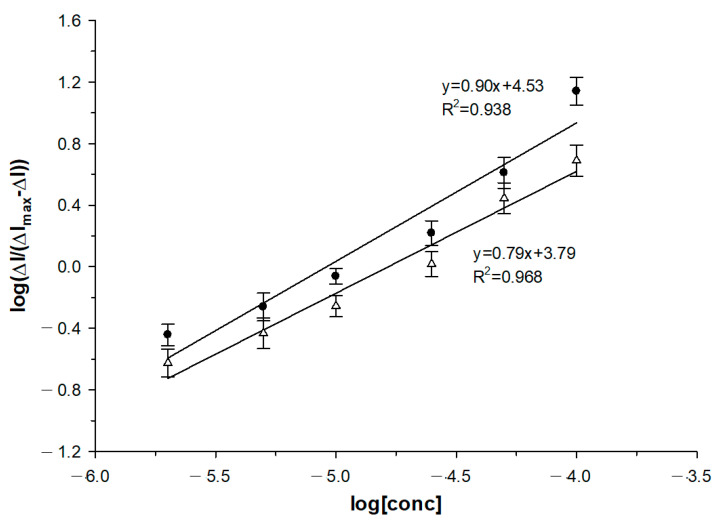
Relationship between log(ΔI/(ΔI_max_ − ΔI)) and log[conc] of myo-inositol (●) and D-chiro-inositol (Δ) using a BSA/MB/MWCNT/SPGE based biosensor.

According to Equation (1), the association constant (*k*) and the binding number (m) can be obtained from the intercept, taking into account the logarithmic relationship, and the slope of the linear plot, respectively. As shown in Figure 7, two straight lines were obtained in the concentration range of 2–100 μM for both stereoisomers. From the intercept of the linear plot for each curve, the *k* values were calculated to be 3.4 × 10^4^ and 6.2 × 10^3^ L mol^−1^, for myo-Ins and D-chiro-Ins, respectively, thus confirming the stronger complexation ability of BSA with myo-Ins. The m values, calculated as the slopes of the linear plots, had results of 0.90 and 0.79, for the myo-Ins and D-chiro-Ins, respectively. The values obtained were close to 1, thus indicating a 1:1 complex when BSA was bound to both stereoisomers, which is in agreement with other results obtained for BSA complexes with different chiral biomolecules [38].

The DPV technique was also employed to assess the sensor stability in the presence of 50 μM of myo-Ins (Figure S5, Supplementary Materials). The modified sensors were kept at 4 °C on different days for 28 days and tested every 7 days. The stability result was very good, with a current response of about 3% of its initial response on day 21 and of 10% on day 28. The biosensor also showed a fast response time of 5 s.

### 3.7. Molecular Docking Studies

Preliminary molecular docking analysis was performed to investigate the BSA–ligand interactions.

**Figure 8:**
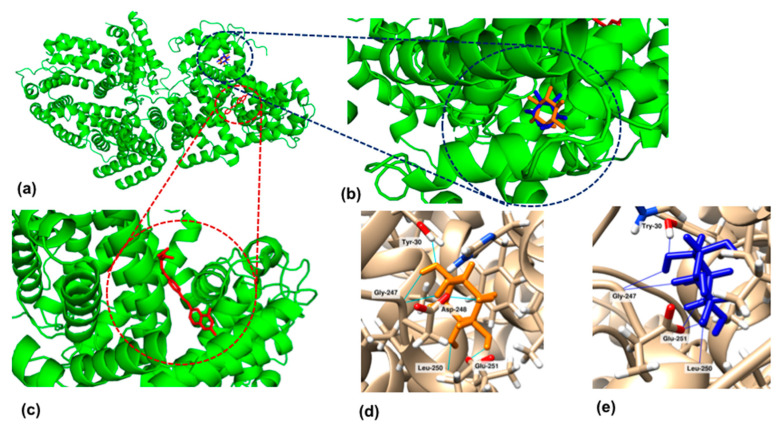
(**a**) Best conformation of whole BSA in carton ribbons with the three ligands: myo-Ins (orange color), D-chiro-Ins (blue color), folic acid (red color); (**b**) zoom of the predicted binding site with myo-Ins and D-chiro-Ins; (**c**) zoom of the predicted binding site with folic acid; (**d**,**e**) zoom of the H-bonds of the predicted binding sites for myo-Ins and D-chiro-Ins, respectively.

Figure 8a,b shows that the myo-Ins (orange) and D-chiro-Ins (blue) were docked at the same site of the BSA.

As reported in Table 3, the myo-Ins show a higher number of predicted sites [36] compared to D-chiro-Ins [29] and a more negative best docking score, which means a more probable binding model. However, the prediction of the binding affinity (ΔG) of the ligands with BSA protein displayed that the myo-Ins and D-chiro-Ins showed the same affinity value (−6.74 kcal/mol), probably because they are stereoisomers, and the predicted binding site is composed of the same amino acid sequence.

Each stereoisomer formed six H-bond interactions, as shown in Figure 8d,e. In particular, myo-Ins formed four H-bonds with four different amino acids, namely Tyr-30, Leu-250, Glu-251, and two H-bonds with the same Gly-247, while D-chiro-Ins formed two H-bonds with two different amino acids, Tyr-30 and Leu-250, and two H-bonds with both Glu-251 and Gly-247.

A third ligand, folic acid, was investigated as a potential interferent present in several commercially available pharmaceutical preparations. It showed a different BSA binding site (Figure 8c), and therefore, a slightly higher ΔG value (−7.31 kcal/mol). This fact could be ascribed to the different structure of folic acid compared to the previous stereoisomers and to the different amino acid sequences of the binding site (two H-bonds with Tyr-156 and Arg-256).

The results obtained with the docking analysis are in good agreement with the association constant values obtained with the electrochemical experiments reported in Section 3.6.

### 3.8. Application of the Stereoselective Biosensor

Combined therapy with myo-Ins and D-chiro-Ins has been found to reduce the risk of metabolic disease in PCOS patients compared to myo-Ins supplementation alone [58]. Therefore, in recent years, several supplements containing the two stereoisomers in different ratios have been produced and commercialized, although the 40:1 myo-Ins/D-chiro-Ins ratio was more effective for PCOS therapy, aimed at restoring ovulation and normalizing different hormone levels.

**Figure 9:**
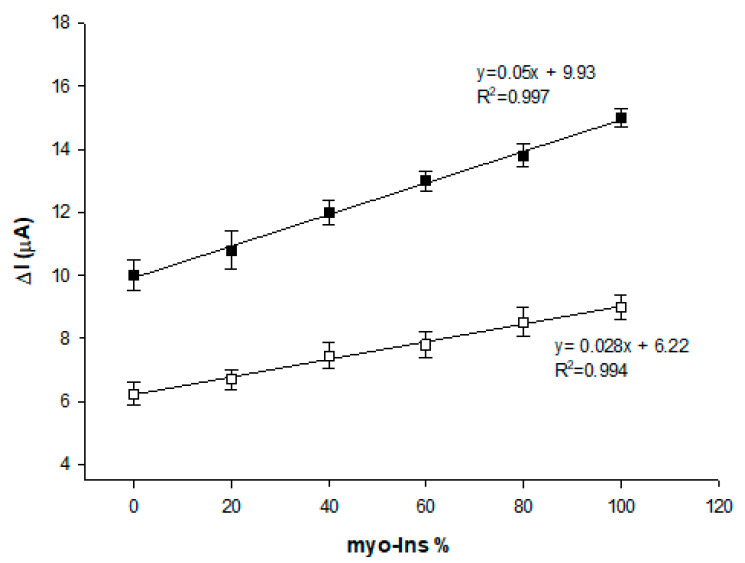
Current response of BSA/MB/MWCNT/SPGE with stereoisomer mixture of myo- and D-chiro-inositols at different total concentrations: (□)10 μM; (■) 50 μM.

Therefore, the possibility of detection of the isomers ratio in mixture solutions is an important issue. The DPV curves of the solutions with different myo-/D-chiro-Ins ratios show progressive decreasing oxidation peaks (and therefore, an increasing ΔI value) at increasing concentrations of myo-Ins (myo-Ins % from 0 to 100%). Figure 9 shows the calibration curves of the BSA/MB/MWCNT/GSPE current response as a function of the myo-/D-chiro-Ins mixture at two different total concentrations of 10 and 50 μM. Good linearity in the range of 0–100% of myo-Ins is observed for both calibration curves, confirming the feasibility of an accurate prediction of the stereoisomer ratio with the developed biosensor.

Lastly, the proposed biosensor was tested for the detection of myo-Ins and D-chiro-Ins in four commercial pharmaceutical preparations. The measurements were repeated five times for each sample, with Table 4 showing the averaged results. It is possible to note that the results obtained with the proposed immunosensor are in good agreement with those claimed by the manufacturers in terms of precision, with RSD values ranging from 1.5 to 3.8% for myo-Ins and from 2.6 to 4.4% for D-chiro-Ins. It is known that the compositions of the commercial drugs, reported in Table S1, can affect the biosensor response. In particular, folic acid, Mg^2+^ and Se^2+^ ions, Vitamin D_3_, α-lattoalbumin, and melatonin are generally present in the pharmaceutical preparations containing myo-Ins and D-Chiro-Ins available on the market. Selectivity studies were performed in order to test the effects of the above-mentioned compounds as possible interferents, by comparing the DPV current signal obtained with myo-Ins and D-Chiro-Ins and all other compounds at a ten-times-higher concentration. The results show that no significative interference was observed for all compounds tested (Figure S6), confirming the potential of the developed biosensor for practical applications [59]. It must be noted that folic acid displayed a slightly stronger BSA affinity binding, as reported in Table 3; however, its concentrations in the pharmaceutical preparations analyzed (Table S1) were much lower compared to the two stereoisomers, and therefore, its interference is negligible.

## 4. Conclusions

A reagentless chiral voltammetric biosensor for the discrimination of myo- and D-chiro-Ins diastereoisomers was successfully developed based on a novel BSA/MB/MWCNT/GSPE modified electrochemical platform. The results confirm that larger current signals were obtained with myo-Ins due to its lower hydrophobic nature compared to D-chiro-Ins, which allows for better matching with the hydrophilic cavities of the BSA binding site. These results were confirmed by computational studies. The linear range of the biosensor was found to be 2–100 μM for both stereoisomers, with a higher sensitivity for myo-Ins compared to D-chiro-Ins. Additionally, the proposed biosensor was successfully utilized for the quantitative analysis of stereoisomeric excess in a myo-Ins/D-chiro-Ins mixture of four commercial pharmaceutical drugs, with adequate precision. This study is helpful not only for comprehending the interactions between BSA and myo-/D-chiro-Ins molecules but also, in general, for understanding the stereoselective interactions between proteins and chiral species, such as chiral and illegal drugs, with important applications in the field of pharmaceutical and forensic sciences for the quality control of drugs during their production stage.

## Figures and Tables

**Table 1:** Potential peak separation (ΔE_p_), heterogeneous electron transfer rate constant (k^0^), real electroactive area (A_EA_), and roughness factor (*ρ*) of a bare and MWCNT/GSPE.

GSPE	ΔE (mV)	k^0^ (10^−3^ cm s^−1^)	A_EA_/(cm^−2^)	*ρ*
Bare	190	1.38 ± 0.16	0.14 ± 0.002	1.29
MWCNTs	105	1.66 ± 0.14	0.20 ± 0.003	1.67

**Table 2:** Fitting parameters obtained from the Randles equivalent circuit of modification steps of GSPE.

GSPE	R_S_ (Ω)	R_CT_ (Ω)	Q (μMho × s^N^)	C (μF)	W (μMho × s^1/2^)
* Bare	153	2.12 × 10^3^	25.5	-	995 × 10^−6^
MWCNTs	182	448	-	7.41	1.15 × 10^−3^
MWCNT/MB	187	142	-	20.3	1.33 × 10^−3^
MWCNT/MB/BSA	201	430	-	8.2	1.25 × 10^−3^

* N = 0.686.

**Table 3:** Docking parameters, ΔG affinity, and amino acid residues involved in the predicted binding site. Abbreviations: NTPS = number of total predicted sites.

Complex with BSA	NTPS	Best Docking Score	ΔG Affinity (Kcal mol^−1^)	Amino Acids in the Vicinity of Ligand
Myo-Ins	36	−120.76	−6.74	Leu-22, Ala-26, Tyr-30 *, Leu-66, His-67, Phe-70, Asn-99, His-246, Gly-247 *, Asp-248 *, Leu-249, Leu-250 *, Glu-251 *
D-chiro-Ins	29	−114.21	−6.74	Leu-22, Val-23, Ala-26, Tyr-30 *, Leu-66, His-67, Phe-70, Asn-99, His-246, Gly-247 *, Asp-248, Leu-249, Leu-250 *, Glu-251 *
Folic acid	100	−175.64	−7.31	Tyr-149, Glu-152, Tyr-155, Tyr-156 *, Lys-159, Lys-187, Thr-190, Ser-191, Arg-194, Arg-198, Leu-237, Val-240, His-241, Arg-256 *, Leu-259, Ser-286, His-287, Ile-289, Ala-290, Glu-291

* Hydrogen bonding with this amino acid.

**Table 4:** Detection of myo-Ins and D-chiro-Ins in real samples (*n* = 5).

Commercial Pharmaceutical Preparation	Stereoisomer	Label Value mg/Tablet	Found mg/Tablet	RSD %
Preparation 1	Myo-Ins	200	202.8 ± 4.5	2.2
	D-chiro-Ins	500	498.5 ± 2.4	2.6
Preparation 2	Myo-Ins	600	597.6 ± 2.0	1.5
	D-chiro-Ins	-	-	2.9
Preparation 3	Myo-Ins	550	553.2 ± 3.3	3.2
	D-chiro-Ins	15	13.9 ± 4.1	2.8
Preparation 4	Myo-Ins	2000	1995.6 ± 3.8	3.8
	D-chiro-Ins	50	46.7 ± 4.5	4.4

## Data Availability

Data are contained within the article.

## Supplementary Materials

The following supporting information can be downloaded at: https://www.mdpi.com/article/10.3390/s23229211/s1, Figure S1: SEM image of bare GSPE. Experimental conditions: magnification: 25 K X; voltage: 1.50 kV.; Figure S2: Raman spectra of bare GSPE (green) and MWCNT/GSPE (red); Figure S3: Scan rate effect of: (a) bare GSPE and (b) MWCNT/GSPE at 5 mV s^−1^ (black line), 10 mV s^−1^ (red line), 25 mV s^−1^ (blue line), 50 mV s^−1^ (green line), and 100 mV s^−1^ (pink line). Redox probe: 5 mM [Fe(CN)_6_]^3−/4−^ containing 0.1 M KCl solution.; Figure S4: Relationship between (a) the volume of BSA and (b) the incubation time of BSA vs. the peak response in PBS 50 mM, pH 7.0, containing 50 µM of myo-inositol. Figure S5: Stability of BSA/MB/MWCNT/GSPE at 4 °C (*n* = 6), in the presence of 50 µM myo-Ins. Figure S6: Histograms of selectivity assay for Myo-Ins vs. different interferent compounds. Experimental conditions: DPV experiments in Zobell’s solution; myo-Ins: 50 μM; all other compounds: 0.5 mM. Table S1: Information about the manufacturer and ingredients of commercial pharmaceutical preparations containing Myo-Ins and D-chiro-Ins.
