# Supplementary material for: Stereoselective Voltammetric Biosensor for Myo-Inositol and D-Chiro-Inositol Recognition
Source: Sensors (Basel). 2023 Nov 16;23(22):9211. doi: 10.3390/s23229211 (PMC10674735; doi:10.3390/s23229211)
Supplement: Supplementary file 1 [file sensors-23-09211-s001.zip › sensors-2684962-supplementary.pdf]

sensors MDPI

Supplementary materials

Stereoselective voltammetric biosensor for myo-inositol and D-chiro-inositol recognition

Cristina Tortolini[a], Valeria Giglia[a], Flavio Rizzo[a], Andrea Lenzi[a], Mariano Bizzarri[a], Antonio Angeloni[a] and Riccarda Antiochia[2b]*

[a]Department of Experimental Medicine, "Sapienza", University of Rome, V.le Regina Elena 324, 00161 Rome, Italy
[b]Department of Chemistry and Drug Technologies, "Sapienza", University of Rome, P.le Aldo Moro 5, 00185 Rome, Italy
* Correspondence:riccarda.antiochia@uniroma1.it (R.Antiochia)

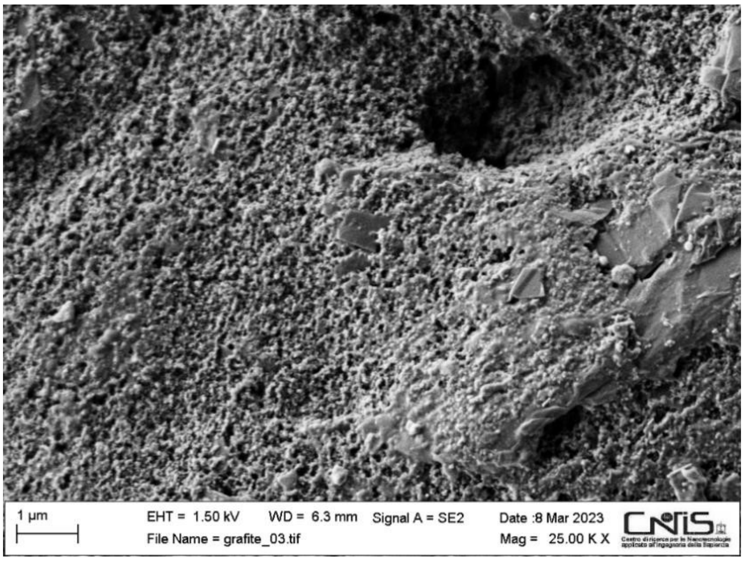

Figure S1. SEM image of bare GSPE. Experimental conditions: magnification 25 K X; voltage 1.50 kV.

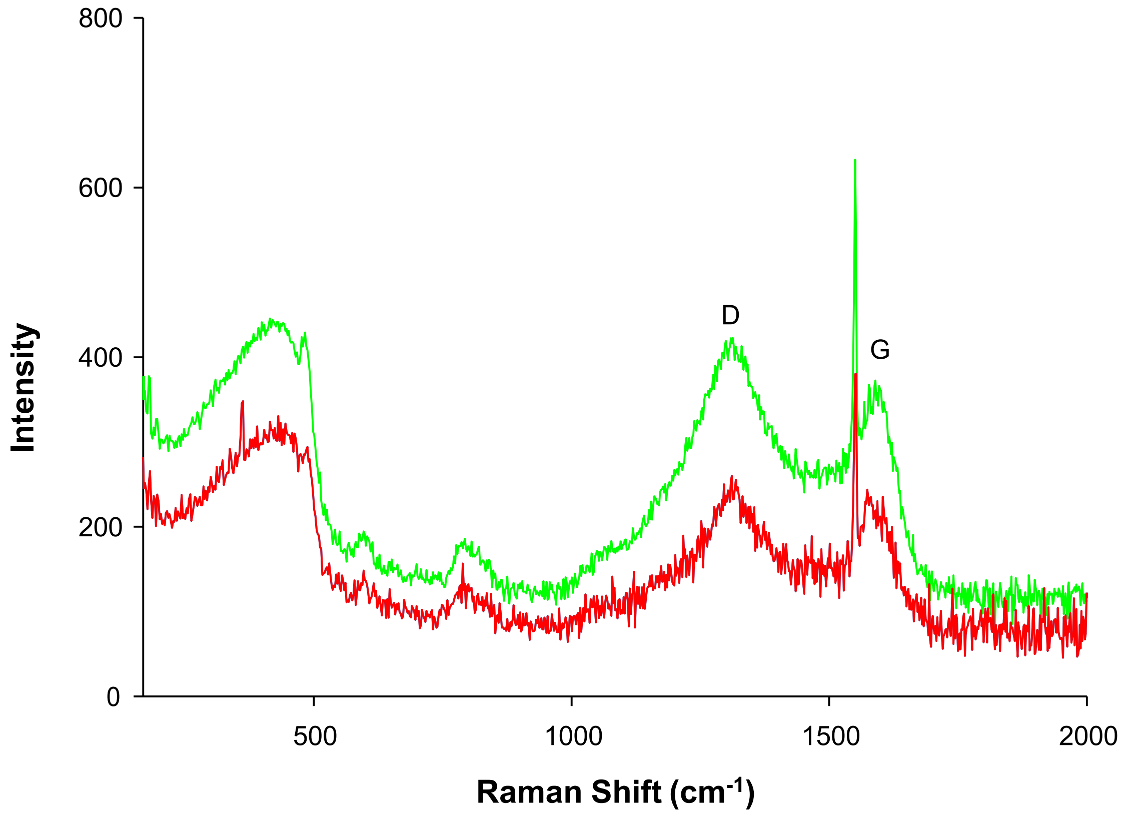

Figure S2. Raman spectra of bare GSPE (green) and MWCNTs/GSPE (red).

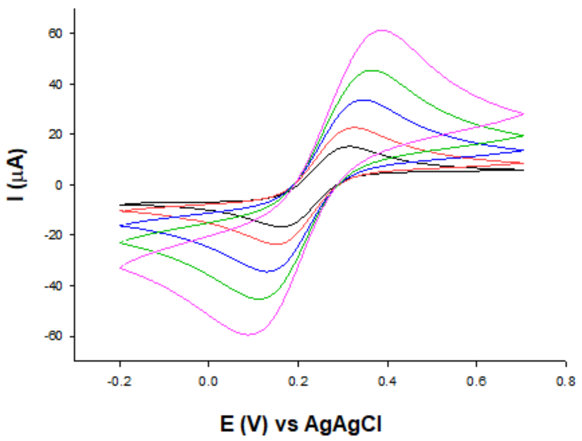

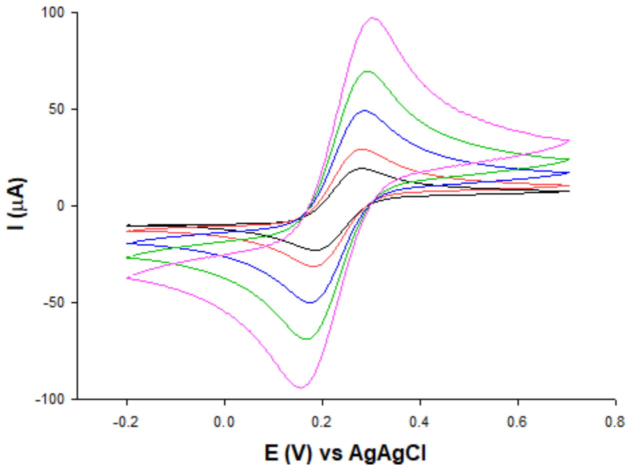

Figure S3. Scan rate effect of: (left) SPGE and (right) MWCNTs/SPGE at 5 mV s^{-1} (black line), 10 mV s^{-1} (red line), 25 mV s^{-1} (blue line), 50 mV s^{-1} (green line) and 100 mV s^{-1} (pink line). Redox probe: 5mM $[Fe(CN)_6]^{3-/4-}$ containing 0.1 M KCl solution.

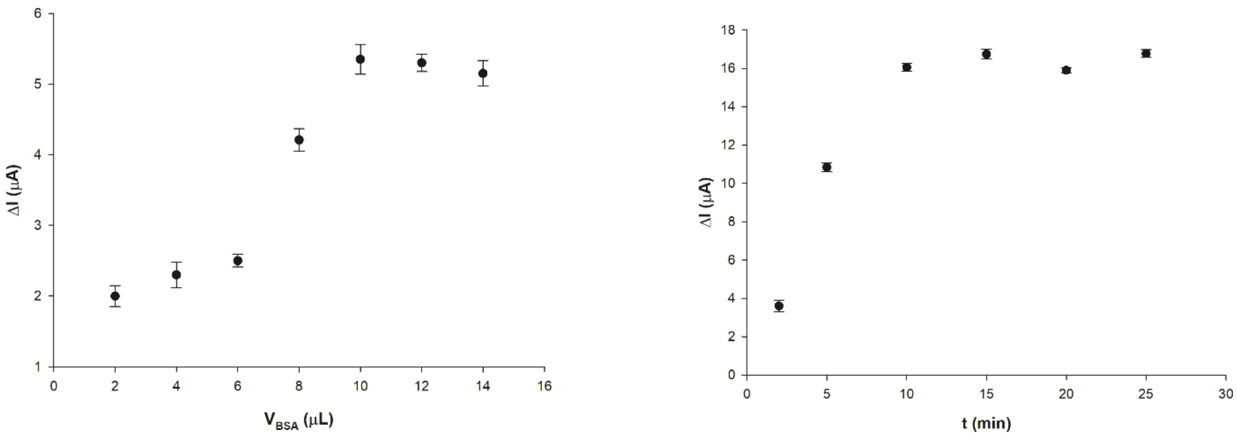

Figure S4. Relationship between (left) the volume of BSA and (right) the incubation time of BSA vs. the peak response in PBS 50 mM, pH 7.0 containing 50 μM of myo-inositol.

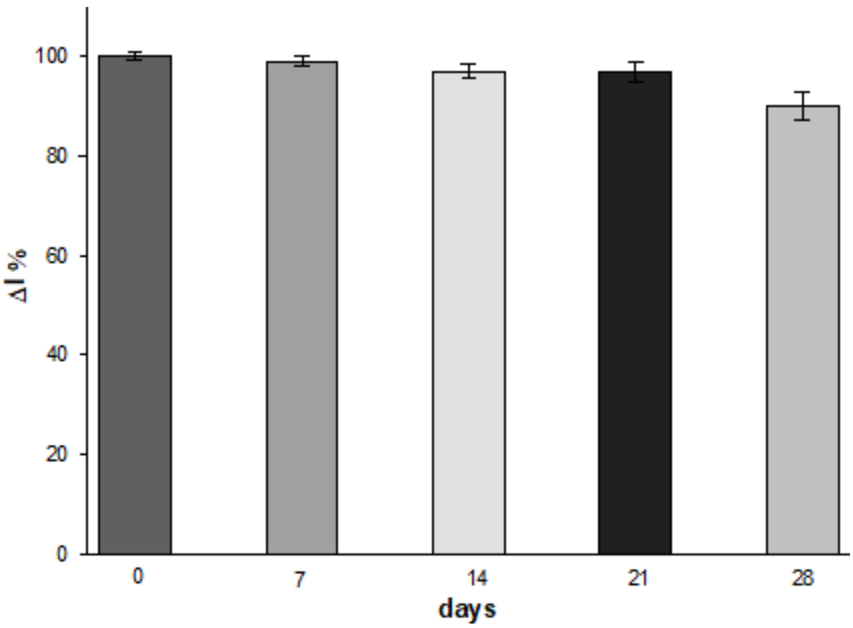

Figure S5. Stability of BSA/MB/MWCNTs/GSPE at 4 °C (n=6), in presence of 50 μM myo-Ins.

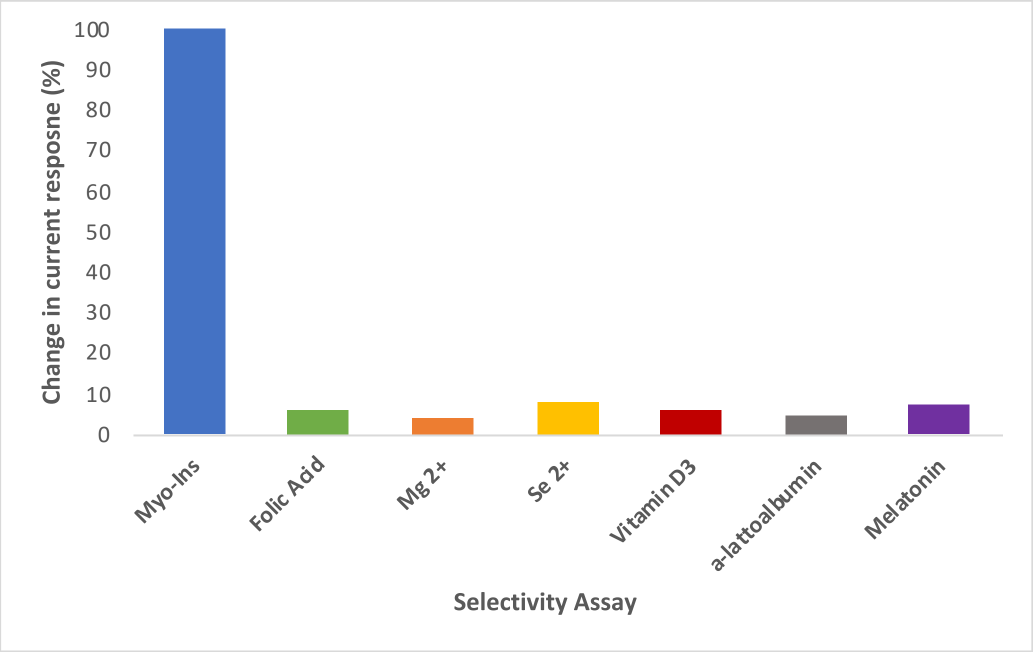

Figure S6. Histograms of selectivity assay for Myo-Ins vs. different interferent compounds. Experimental conditions: DPV experiments in Zobell's solution; myo-Ins: 50 µM; all other compounds: 0.5 mM.

Table S1. Information about the manufacturer and ingredients of commercial pharmaceutical preparations containing Myo-Ins and D-chiro-Ins.

Pharmaceutical preparation	Commercial name and Manufacturer	Ingredients (for 1 capsule)
Preparation 1	Kirocomplex, S& R Pharmaceutics	D-Chiro-Ins 500 mg<br>Myo-Ins 200 mg<br>Mg^{2+} 5 mg<br>Folic Acid 200 μg<br>Vitamin D_3 12.5 μg
Preparation 2	Tiroxil 4.0, Lo.Li Pharma S.r.l.	Myo-Ins 600 mg<br>Se^{2+} 83 μg
Preparation 3	Inofert Combi Hp, Italfarmaco S.p.A.	Myo-Ins 550 mg<br>D-chiro-Ins 15 mg<br>Folic Acid 200 μg
Preparation 4	Eufertix, Pharmaspeed	α-Lattoalbumin 14.1 mg<br>Myo-Ins 2000 mg<br>D-chiro-Ins 50 mg<br>Folic Acid 400 μg<br>Melatonin 1 mg
